# Genomic Instability and Cellular Senescence: Lessons From the Budding Yeast

**DOI:** 10.3389/fcell.2020.619126

**Published:** 2021-01-12

**Authors:** Jee Whu Lee, Eugene Boon Beng Ong

**Affiliations:** ^1^Institute for Research in Molecular Medicine (INFORMM), Universiti Sains Malaysia, Penang, Malaysia; ^2^USM-RIKEN International Centre for Aging Science (URICAS), Universiti Sains Malaysia, Penang, Malaysia

**Keywords:** aging, longevity, rDNA stability, *Saccharomyces cerevisiae*, senescence, telomere length homeostasis

## Abstract

Aging is a complex biological process that occurs in all living organisms. Aging is initiated by the gradual accumulation of biomolecular damage in cells leading to the loss of cellular function and ultimately death. Cellular senescence is one such pathway that leads to aging. The accumulation of nucleic acid damage and genetic alterations that activate permanent cell-cycle arrest triggers the process of senescence. Cellular senescence can result from telomere erosion and ribosomal DNA instability. In this review, we summarize the molecular mechanisms of telomere length homeostasis and ribosomal DNA stability, and describe how these mechanisms are linked to cellular senescence and longevity through lessons learned from budding yeast.

## Introduction

Mammalian cells possess a finite replicative capacity known as “Hayflick limit” (Hayflick and Moorhead, 1961) which when reached initiates replicative senescence upon irreversible cell cycle arrest (Demidenko and Blagosklonny, 2008). Replicative senescence is a natural process that occurs due to telomere shortening with every cell division (Stewart et al., 2003). However, premature senescence can be induced by cellular exposure to stresses such as oxidative stress or DNA damage (Debacq-Chainiaux et al., 2016). Cellular senescence is thought to be a beneficial protective system against cancer because it limits the proliferation of damaged cells and progression of malignant cells (Muñoz-Espín and Serrano, 2014). Senescent cells remain metabolically active and are viable for a long period of time (Blagosklonny, 2003), and exhibit phenotypes such as enlarged intracellular organelles and increased cell size (Cristofalo et al., 2004; Matsui and Matsuura, 2010)

While aging is caused by structural deteriorations at the organismal level, at the cellular level aging is caused by replicative or premature senescence *via* genomic instability amongst other factors (Lidzbarsky et al., 2018; Lagunas-Rangel and Bermúdez-Cruz, 2019). In this mini review, we summarize our current understanding of telomere length homeostasis and maintenance of ribosomal DNA (rDNA) stability, the two major contributors to genomic instability.

## Maintenance of Telomre Length Homeostasis Prevents Cellular Senescence and Aging

**FIGURE 1:**
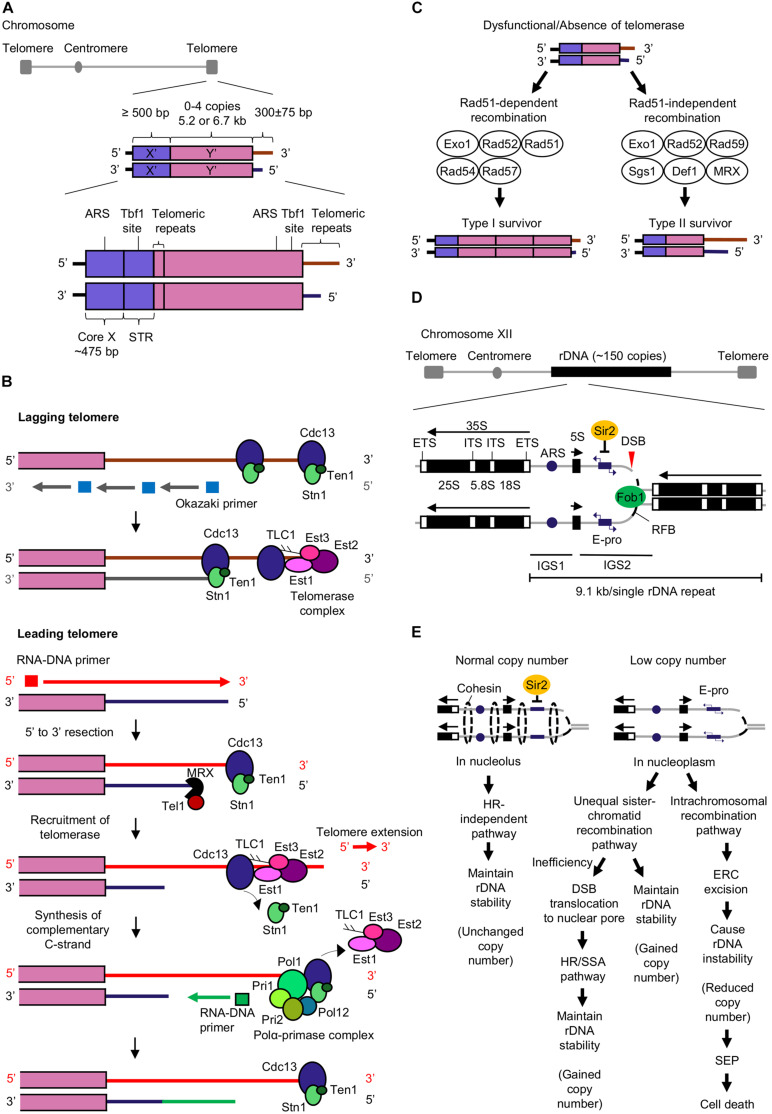
Mechanisms of telomere extension and rDNA copy number maintenance that prevent cellular senescence and aging. **(A)** The budding yeast telomere structure consists of X′ and Y′ elements, and telomeric repeats. Core X that contains an autonomously replicating sequence (ARS) which is an origin of replication, and subtelomeric repeated elements (STR) that contains a Tbf1 binding site, are found in the X′ element. The ARS and Tbf1 binding site are also found in the Y′ element (Louis et al., 1994; Tham and Zakian, 2002; Wellinger and Zakian, 2012). **(B)** Telomerase-dependent pathway for telomere extension. Cdc13 and telomerase complex (Est1, Est2, Est3, and Tlc1) bind to both leading and lagging telomeres (Faure et al., 2010). DNA polymerase α (Polα)-primase complex generates RNA-DNA primers that initiate the synthesis of Okazaki fragments by DNA polymerase δ (Pol δ) (McElhinny et al., 2008; Perera et al., 2013) at lagging strand. After removal of the primers, the Okazaki fragments are ligated by DNA ligase I to form complementary lagging strand (Faure et al., 2010; Perera et al., 2013; Liu et al., 2017). Telomere extension mediated by telomerase occurs primarily at the leading telomere (Faure et al., 2010). The RNA-DNA primer is required for initiating synthesis of complementary leading strand by DNA polymerase ε (Pol ε) (Pursell et al., 2007; McElhinny et al., 2008; Perera et al., 2013). CST complex (Cdc13-Stn1-Ten1) bound at telomere end restricts the access of telomerase to telomere end. MRX complex (Mre11-Rad50-Xrs2) induces the binding of Tel1 (Nakada et al., 2003; McGee et al., 2010) to short telomere and executes 5′ to 3′ exonuclease activity to synthesize 3′ overhang (Diede and Gottschling, 2001). Both MRX and Tel1 promote Cdc13-mediated telomerase tethering to telomere (Tseng et al., 2006; Yang et al., 2017). Extension of telomere by telomerase is completed upon the synthesis of the CST complex (Pfeiffer and Lingner, 2013) and telomerase departure from the telomere. Polα-primase complex (Pol1, Pol12, Pri1, and Pri2) (Lue et al., 2014) interacts with CST complex at the telomere (Churikov et al., 2013) and generates an RNA-DNA primer for synthesis of complementary C-strand (Churikov et al., 2013; Pfeiffer and Lingner, 2013). **(C)** Homologous recombination pathways for telomere extension in the absence of telomerase or when telomerase is dysfunctional. The Rad51-dependent recombination pathway for the generation of type I survivors requires Exo1, Rad52 (Chen et al., 2001; Maringele and Lydall, 2004), Rad51, Rad54, and Rad57 (Chen et al., 2001; Claussin and Chang, 2015) while the Rad51-independent recombination pathway for the generation of type II survivors includes Exo1, Rad52 (Chen et al., 2001; Maringele and Lydall, 2004), Rad59, Sgs1, Def1, and MRX complex (Huang et al., 2001; Signon et al., 2001; Chen et al., 2005). **(D)** The structure of budding yeast rDNA (Poveda et al., 2010; Chand Dakal et al., 2016; Kobayashi and Sasaki, 2017). Abbreviations: ETS, external transcribed spacer; ITS, internal transcribed spacer; IGS, intergenic spacer; ARS, autonomous replication sequence; E-pro, rDNA non-coding promoter; RFB, replication fork barrier site; DSB, double-stranded DNA break. **(E)** Regulation of rDNA stability by homologous recombination (HR)-independent and -dependent pathways (unequal sister-chromatid or intrachromosomal recombination pathway) (Kobayashi and Sasaki, 2017; Sasaki and Kobayashi, 2017; Horigome et al., 2019; Morlot et al., 2019). Abbreviations: DSB, double-stranded break; ERC, extrachromosomal rDNA circles; SEP, senescence entry point; SSA, single strand annealing.

Telomeres at eukaryotic chromosome ends protect the chromosome ends from end-to-end fusion, degradation, and prevent misrecognition of the ends as double-stranded DNA breaks (DSBs) (Tham and Zakian, 2002; Dieckmann et al., 2016). Telomeres have terminal single-stranded (ss) DNA overhangs with 3′ repetitive guanine-rich sequences (termed G tail or G-overhang) (Giraud-Panis et al., 2010; Eugène et al., 2017). Telomeres are marked by tandem repeats such as G_3_T_2_A in vertebrates and TG_1__–__3_ in budding yeast *Saccharomyces cerevisiae* (Tran et al., 2011; Wellinger and Zakian, 2012). In budding yeast, telomeres consist of subtelomeric repeats known as X′ element found in all telomeres and Y′ element found in two-thirds of telomeres (Louis and Haber, 1992; Teng and Zakian, 1999; Figure 1A).

### Telomere Shortening

Telomere shortening (or erosion) causes genomic instability through the breakage-fusion-bridge cycle. The progressive loss of telomere end after cell division (Soudet et al., 2014) initiates DNA break repair (DBR) machinery that repairs shortened telomeres through DNA replication creating a fusion of two sister chromatids. During cell division, the segregation of fused chromosomes will cause a random break, leading to inheritance of deleted or amplified chromosomes by daughter cells. The continuous breakage-fusion-bridge cycle with every cell division leads to genomic instability (McClintock, 1938; Tanaka and Yao, 2009).

### Telomere Length Homeostasis

Lagging and leading telomeres are synthesized in the progression of replication fork during telomere replication. Due to the end-replication problem, telomeres are replicated incompletely by DNA polymerases and end with a 3′ overhang. Excessive critically short telomeres will elicit a DNA damage signal causing permanent cell cycle arrest, subsequently cellular senescence and death (Shay and Wright, 2005; Aubert and Lansdorp, 2008).

Additionally, dysfunctional telomeres due to telomere uncapping can cause cellular senescence in an indirect manner. The unprotected telomeres undergo degradation (Vodenicharov and Wellinger, 2006; Ghadaouia et al., 2018) and induce a weak DNA damage response (DDR). Unprotected telomeres are prone to chromosomal end fusion resulting in secondary DNA breaks and genomic instability, eliciting a strong DDR. Consequently, the additional DNA damage causes permanent growth arrest and cellular senescence (Ghadaouia et al., 2018). Therefore, telomere length homeostasis must be maintained by telomere elongation to compensate for the end-replication problem and protect telomere ends. The two pathways involved in telomere elongation are telomerase-dependent pathway and homologous recombination (HR) pathway.

### Telomerase-Dependent Pathway

Telomerase is a reverse transcriptase that depends on its internal RNA subunit Tlc1 (Gilson and Géli, 2007) as a template to extend telomeric repeats. Telomerase preferentially extends short telomeres in the late S phase (Wellinger and Zakian, 2012) by applying dNTP synthesized by ribonucleotide reductase (RNR) to add nucleotides at the telomeric 3′ overhang (Maicher et al., 2017) while the complementary strand is synthesized by DNA polymerases (Hug and Lingner, 2006; Figure 1B). A telomerase consists of four subunits Est1, Est2, Est3, and Tlc1 which positively regulate telomerase activity for telomere extension (Lendvay et al., 1996). Est1 enables the access of telomerase to telomere by interacting with Tlc1 and telomeric ssDNA-binding protein Cdc13 (Virta-Pearlman et al., 1996; Evans and Lundblad, 1999; Zhou et al., 2000; Li et al., 2013). Additionally, Est1 stimulates the generation of G-quadruplex at telomeric overhang for telomere extension and protection (Zhang et al., 2010; Tong et al., 2011; Li et al., 2013). Est2 (Counter et al., 1997) and Tlc1 (Singer and Gottschling, 1994; Cohn and Blackburn, 1995) catalyze telomere extension. Est3 which associates with Est1 and Est2, induces Est2’s catalytic activity for telomere extension (Zhang et al., 2010; Mariasina et al., 2018).

### Telomerase-Independent Homologous Recombination Pathway

Telomerase deficiency leads to progressive telomere shortening, and consequently cell death (Le et al., 1999). Nevertheless, a subset of cells with telomerase deficiency can still survive and have extended telomeres. These survivors are classified as type I or type II; with their telomeres extended *via* Rad51-dependent or Rad51-independent homologous recombination (HR) pathways respectively. Both recombination pathways involve Rad52, Exo1, and Pol32 which is a non-essential subunit of DNA polymerase δ (Chen et al., 2001; Maringele and Lydall, 2004; Lydeard et al., 2007; Figure 1C).

The telomeres of type I survivors contain tandemly amplified Y′ elements and short telomeric repeats TG_1__–__3_ at their ends while telomeres of type II survivor have amplified telomeric repeats TG_1__–__3_ at their ends with heterogenous length (Figure 1C). Although the survivors can depend on HR pathway to maintain telomere length, they possess shorter replicative life spans (RLS). The reactivation of telomerase activity can restore the reduced RLS, revealing the role of telomerase in sustaining cellular RLS possibly by suppressing telomere recombination and maintaining telomere length (Chen et al., 2009). Additionally, other proteins involved in the regulation of telomere length homeostasis in *S. cerevisiae* are summarized in Table 1.

**TABLE 1:** Functions of proteins involved in the regulation of telomere length homeostasis in *Saccharomyces cerevisiae*.

**Proteins**		**Functions**
**Telomere capping complexes:**		
Cdc13-Stn1-Ten1	CST complex	Protects telomere end from degradation (Grandin et al., 2001) Restricts telomerase access to telomere at the end of S phase (Churikov et al., 2013).
Ku70-Ku80	Yku complex	Protects telomere end from telomere-end resection (Vodenicharov et al., 2010; Shi et al., 2013).
Rap1-Rif1-Rif2		Restricts telomerase access to telomere to inhibit telomere extension of overlengthened telomere (Wotton and Shore, 1997; Goudsouzian et al., 2006; Hirano et al., 2009). Inhibits exonucleolytic degradation of telomere by preventing the association of Mre11-Rad50-Xrs2 (MRX) complex with telomere (Bonetti et al., 2010).
**Telomere capping proteins:**		
Npl3, Cdc2	hnRNP-related proteins	Prevents telomere end from being recognized as DNA break (Lee-Soety et al., 2012)
Rad6–Bre1–H2Bub1		Promotes telomere extension by inducing telomere-end resection (Wu et al., 2017)
Mre11-Rad50-Xrs2	MRX complex	Executes 5′ to 3′ exonuclease activity to synthesize 3′ overhang for Cdc13 binding at telomere (Diede and Gottschling, 2001) Mediates telomerase tethering to telomere (Tsukamoto et al., 2001). Protects uncapped telomere from exonucleolytic degradation during telomere extension (Vodenicharov and Wellinger, 2007; Wu et al., 2018).
Cdc13	ssDNA-binding protein	Protects telomere end (Nugent et al., 1996; Pennock et al., 2001). Promotes telomerase tethering to telomere (Nugent et al., 1996; Chandra et al., 2001)
Mec1	Phosphoinositide 3-kinase-related kinases (PIKKs)	Phosphorylates Cdc13 to mediate telomerase tethering to telomere (Tseng et al., 2006; Yang et al., 2017). Phosphorylates Rap1 to strengthen the interaction of Rap1 with Rif1 for promoting telomere end protection (Yang et al., 2017).
Tel1	Phosphoinositide 3-kinase-related kinases (PIKKs)	Recruited by MRX complex to short telomere (Nakada et al., 2003, 1; McGee et al., 2010) to promote telomerase tethering to telomere (Goudsouzian et al., 2006; Sabourin et al., 2007). Phosphorylates Cdc13 to mediate telomerase tethering to telomere (Tseng et al., 2006; Yang et al., 2017). Phosphorylates Rap1 to strengthen the interaction of Rap1 with Rif1 to promote telomere end protection (Yang et al., 2017).
Mre11	Double-strand break repair protein	Promotes telomerase tethering to telomere (Goudsouzian et al., 2006).
RPA	ssDNA-binding protein (replication protein A)	Promotes telomerase activity during telomere extension (Schramke et al., 2004; Luciano et al., 2012).
Cdk1	Cyclin-dependent kinase	Regulates telomere extension (Frank et al., 2006).
Pif1	Helicase	Unwinds G-quadruplex at telomere end to enable telomerase-mediated telomere extension and avoid DNA break (Paeschke et al., 2011)
Sgs1	Helicase	Unwinds G-quadruplex at telomere end to enable telomerase-mediated telomere extension (Huber et al., 2002). Generates type II telomerase-deficient survivors (Huang et al., 2001).
Elo3	Fatty acid elongase	Synthesizes very long-chain fatty acids (VLCFAs) (Kvam et al., 2005) to maintain telomere length through mediation of Yku (Ponnusamy et al., 2008).
Kcs1	Inositol hexakisphosphate and inositol heptakisphosphate kinase	Synthesizes inositol phosphates, which negatively affect telomere-maintaining role of Elo3 through mediation of Yku (Ponnusamy et al., 2008).
Ipk2	Inositol polyphosphate multikinase	
Sit4	Protein serine/threonine phosphatase	Synthesizes protein phosphatase 2A (PP2A) which dephosphorylates Sir3 to maintain the heterochromatin structure for telomere stabilization (Chan and Blackburn, 2002; Hayashi et al., 2005).
Def1	RNA polymerase II degradation factor	Positive regulator in telomere maintenance and required for the generation of type II telomerase-deficient survivors (Chen et al., 2005).
Tsa1	Thioredoxin peroxidase	Major reactive oxygen species (ROS) scavenger (Iraqui et al., 2009) that prevents telomere overextension due to ROS (Lu et al., 2013).
Rnr1	Major subunit of ribonucleotide reductase (RNR)	Provides precursors for synthesis of deoxynucleoside triphosphates (dNTPs) required for telomerase-mediated telomere extension (Maicher et al., 2017).
ESCRT-0, –I,–II, and –III	Endosomal sorting complex required for transport (ESCRT)	Maintains telomere length by participating in telomerase-dependent telomere extension (Dieckmann et al., 2016).
Pol ϵ and Pol δ	DNA polymerase	Maintain telomere length (Ohya et al., 2002) by synthesizing chromosomal DNA strands (McElhinny et al., 2008) Exhibits 3′-5′ exonuclease activity for telomeric ssDNA repair during cell cycle arrest (Ohya et al., 2002; Henninger and Pursell, 2014).
Pol α-primase complex		Synthesizes RNA-DNA primer required for synthesis of chromosomal DNA strands (Churikov et al., 2013; Pfeiffer and Lingner, 2013).
Yra1	RNA-binding protein required for mRNA export from nucleus	Overexpression causes telomere shortening (Gavaldá et al., 2016).

### Telomere Shortening and Its Effects on Aging

Telomere shortening decreases life span in mice and humans (Muñoz-Lorente et al., 2019; Whittemore et al., 2019). In mice, reactivation of telomerase activity can rescue premature aging phenotype through restoration of short telomere length and its function (Samper et al., 2001). Telomerase overexpression which promotes telomere extension can prolong life span in mice (Bernardes de Jesus et al., 2012). Furthermore, mice with overlengthened telomeres exhibited less DNA damage, less metabolic aging and an increased life span (Muñoz-Lorente et al., 2019). These findings show that the telomerase-mediated telomere extension can promote life span extension in animal models. Interestingly, telomerase overexpression in mice also reduced cancer incidences (Bernardes de Jesus et al., 2012; Muñoz-Lorente et al., 2019). This highlights the complex link between senescence and cancer suppression because senescence is thought to be a safeguard against cancer.

Overlengthened telomeres has no impact on yeast chronological life span (CLS) (Harari et al., 2017). Conversely, telomere shortening causes RLS extension in budding yeast, possibly due to the relocalization of Sir2/3/4 complex to non-telomeric sites for heterochromatin structure maintenance and increased genomic stability (Austriaco and Guarente, 1997; Liu et al., 2019). Sir2 inhibits extreme CLS extension in yeast (Fabrizio et al., 2005), unlike its importance for RLS maintenance (Kaeberlein et al., 1999). Hence, the opposite roles of Sir2 in CLS and RLS may explain the different effects of telomere length on yeast aging. Furthermore, yeast telomeres do not shorten with age like the mammalian phenotype (D’Mello and Jazwinski, 1991). Therefore, while yeast is useful for telomere maintenance mechanism studies, it may not be the best model to study the telomere-aging link.

## Maintenance of rDNA Stability Prevents Cellular Senescence and Replicative Aging

The ribosomal RNA gene (rDNA) is the most abundant RNA gene that encodes for ribosomal RNA. Ribosomal RNA is essential to form ribosomes with ribosomal proteins for protein synthesis (Kobayashi, 2008). *S. cerevisiae* has ∼150 tandem repeats of a 9.1 kb rDNA unit (Ganley and Kobayashi, 2014), found on chromosome XII locus (Petes, 1979; Figure 1D). These rDNA repeats are the most prone to DNA damage by external (ultraviolet light) and internal factors (replication errors and ROS), causing regional genomic instability. According to rDNA theory of aging, rDNA instability induces an aging signal that triggers DDR and initiate cellular senescence thus limiting cellular life span (Kobayashi, 2008, 2011a, 2014).

### Maintenance of rDNA Copy Number for rDNA Stability

In yeast, Fob1 and Sir2 are major regulators of the rDNA maintenance system. Fob1 binds at the replication fork barrier (RFB) site in rDNA repeat to block the progression of the replication fork to the opposite direction of 35S rRNA gene transcription and form a DSB at the blocking site (Kobayashi, 2003). The blockage of the replication fork initiates the DBR either through a HR-independent or -dependent pathway depending on cellular rDNA copy number (Kobayashi and Sasaki, 2017; Sasaki and Kobayashi, 2017; Figure 1E).

The histone deacetylase Sir2 inhibits E-pro transcription when rDNA copy number is maintained at wild-type level. The inhibited E-pro transcription strengthens cohesion association to the broken DNA end and enables DBR which is independent of HR to occur in nucleolus (Saka et al., 2016; Sasaki and Kobayashi, 2017; Horigome et al., 2019). As a result, this repair leads to an unchanged rDNA copy number, maintaining rDNA stability and generating little to no aging signal (Saka et al., 2016).

In contrast, Sir2 does not inhibit E-pro transcription when rDNA copy number is less than wild-type level. The active transcription of E-pro dissociates the cohesins from the broken DNA end, stimulating the unequal sister chromatid recombination to occur between the misaligned rDNA repeats for DBR. The unequal sister chromatid recombination is Rad52-dependent (Torres-Rosell et al., 2007) and occurs in nucleoplasm (Horigome et al., 2019). As a result, unequal sister chromatid recombination duplicates and restore rDNA copy number (Kobayashi and Sasaki, 2017).

However, when the unequal sister chromatid recombination mediated DBR is inefficient, the rDNA break ends move to the nuclear pore and interact with nuclear pore complex. These DSBs at the nuclear pore may be repaired either by the HR or single strand annealing pathway. DNA damage checkpoint-associated Mec1/Tel1 kinases, replisome component Tof1 and proteins Tof2, Csm1, and Lrs4 that tether condensins to rDNA assist in the translocation of DSBs to nuclear pore for DBR and maintain rDNA stability (Horigome et al., 2019).

Intrachromosomal recombination can also occur upon activation of E-pro transcription, whereby the broken DNA end recombines with the rDNA copy within the same chromosome. Intrachromosomal recombination is mediated by Rad52 (Park et al., 1999) and results in the formation of extrachromosomal rDNA circles (ERCs) which are segregated from rDNA strand, leading to rDNA copy loss (Kobayashi and Sasaki, 2017). The loss of rDNA copy causes rDNA instability and stimulates cellular senescence (Hein et al., 2012; Saka et al., 2016; Figure 1E).

### rDNA Stability and Its Effects on Aging

Extrachromosomal rDNA circles were previously shown to cause aging in yeast and speculated to be the molecular cause of aging in higher species, including mammals (Sinclair and Guarente, 1997). This ERC theory of aging was later disputed by the rDNA theory of aging (Kobayashi, 2008) which affirms that rDNA instability is a major cause of aging independent of ERC accumulation level. Although the accumulation of ERCs and other episomes (plasmids) can shorten yeast RLS, they stimulate rDNA instability, reaffirming rDNA stability as a major life span-determinant (Falcón and Aris, 2003; Ganley et al., 2009; Saka et al., 2013). Nevertheless, ERCs can be markers for rDNA instability.

Asymmetrical segregations of instable rDNAs and ERCs occur more frequently to yeast mother cells, resulting in cellular senescence and aging while stable rDNAs are segregated to daughter cells, allowing daughter cells to undergo rejuvenation (Kobayashi, 2011b; Morlot et al., 2019). However, rejuvenation of daughter cells produced by old mother cells (after their first 40% of RLS) would be affected, thus exhibiting decreased RLS. This is likely due to impaired asymmetrical segregation of aging factors which are constrained as well in daughter cells (Kennedy et al., 1994).

More recently, Morlot et al. (2019) proposed a model that links the accumulation of ERCs to senescence and longevity. ERC-linked senescence is categorized into three stages: ERC excision, ERC self-replication, and post-SEP interval. Extensive ERC excision from rDNA during intrachromosomal recombination and self-replication of ERC leads to the accumulation of ERC. Even though accumulating ERC can upregulate rDNA transcription, ribosome synthesis is not enhanced. Loss of coordination between rDNA transcription and ribosome biogenesis could negatively affect cell growth. When the amount of ERCs reaches a threshold, the cells reach a senescence entry point (SEP). In post-SEP interval (an interval between SEP and cell death), the cells experience a loss of nuclear homeostasis [an increase in nucleus-to-cell area ratio (N/C ratio), increase in histone content and genomic defect] which causes cell death (Morlot et al., 2019; Figure 1E). The relationship between rDNA stability to RLS has been shown with yeast mutants of rDNA stability regulators. Yeast cells lacking *SIR2* with more ERCs causing rDNA instability exhibited a ∼50% decrease in RLS (Kaeberlein et al., 1999). Unlike *SIR2* mutants, *FOB1* mutants has less ERCs, thus enhancing rDNA stability and showed a 70% extension of RLS (Defossez et al., 1999). More recently, a new regulator of rDNA copy number, Eaf3 was discovered (Wakatsuki et al., 2019). Yeast cells lacking Eaf3 exhibited less ERCs which enhances rDNA stability leading to a 30% extension of RLS. Eaf3 likely activates transcription of E-pro to induce unequal sister chromatid recombination and intrachromosomal recombination which results in the formation of ERCs.

The link of rDNA stability to senescence and aging has also been established in mammalian cell studies. For example, genomic imaging revealed rDNA copy loss in senescent human cells and blood from aged individuals, validating the link between human aging and rDNA instability (Ren et al., 2017). The mammalian Sir2 homolog SIRT7 was also found to function like Sir2 to prevent rDNA instability and consequently cellular senescence *via* chromatin silencing (Paredes et al., 2018).

In summary, DNA replication-based telomere elongation and DBR-based rDNA copy number maintenance are fundamental mechanisms that minimize DNA loss and damage for maintenance of genomic stability, thus inhibiting the onset of cellular senescence and aging. Studies in yeast have identified proteins regulating telomere length homeostasis and the understanding of rDNA copy number maintenance. Current knowledge shows that rDNA instability possibly plays a bigger role than ERCs in aging in yeast and especially higher organisms. Still, yeast will continue to serve as a versatile model for studying rDNA instability and telomere length maintenance mechanisms.

## Author Contributions

JL drafted the manuscript. EO revised and edited further. Both authors approved the final version of the manuscript for submission.

## Conflict of Interest

The authors declare that the research was conducted in the absence of any commercial or financial relationships that could be construed as a potential conflict of interest.
